# Awareness of venous thromboembolism and thromboprophylaxis among hospitalized patients: a cross-sectional study

**DOI:** 10.1186/s12959-017-0144-2

**Published:** 2017-07-19

**Authors:** Hind Almodaimegh, Lama Alfehaid, Nada Alsuhebany, Rami Bustami, Shmylan Alharbi, Abdulmalik Alkatheri, Abdulkareem Albekairy

**Affiliations:** 1 King Abdullah International Medical Research Center/King Saud Bin Abdulaziz University for Health Sciences, College of Pharmacy, Ministry of National Guard Health Affairs, PO BOX 22490, Riyadh, 11426 Saudi Arabia; 2 Pharmaceutical Care Department, King Abdulaziz Medical City, King Abdullah International Medical Research Center/King Saud Bin Abdulaziz University for Health Sciences, PO BOX 22490, Riyadh, 11426 Saudi Arabia

**Keywords:** Venous thromboembolism, Deep vein thrombosis, Pulmonary embolism, Patient awareness, Patient safety

## Abstract

**Background:**

Patient awareness of venous thromboembolism (VTE) and thromboprophylaxis is essential for their safety. In this study, we evaluated patients’ awareness of VTE and their perceptions of thromboprophylaxis.

**Methods:**

We administered a cross-sectional survey to patients hospitalized at the King Abdulaziz Medical City, Riyadh, Saudi Arabia.

**Results:**

Of 190 patients approached, 174 completed the survey, constituting a response rate of 95%. Most participants (72%) were receiving thromboprophylaxis. However, only 32 and 15% reported knowledge of deep vein thrombosis (DVT) and pulmonary embolism (PE), respectively. Fifty-five percent of participants with knowledge of DVT identified swelling of the leg as a symptom. Risk factors for blood clot development were correctly identified by about half of participants, although most agreed that blood clots can cause death (77%). The level of awareness of DVT or PE did not significantly differ by respondents’ demographics. However, awareness of DVT or PE was significantly higher among those with a personal or family history of VTE. Participants had positive perceptions of thromboprophylaxis and were satisfied with treatment (> 69%), but perceived its adverse effects less favorably and reported lower satisfaction with the information provided about DVT and PE (46%).

**Conclusion:**

This study demonstrates the lack of awareness of VTE, DVT, and PE among hospitalized patients. More attention must be paid to patient education to ensure safe and high-quality patient care.

## Key points

This paper shows that there is a lack of awareness about thromboembolism among hospitalized patients that emphasizes the need for improved education in at-risk patients.

## Background

Venous thromboembolism (VTE) is the inappropriate formation of a blood clot in a vein. It can affect hospitalized and non-hospitalized patients, and is associated with considerable mortality, morbidity, and costs [1, 2]. VTE is preventable, but the condition is also unpredictable, with few warning signs. Studies have shown that about 60–70% of cases of deep vein thrombosis (DVT) are clinically undiagnosed and only detected during autopsy. Similarly, at least 70% of cases of fatal pulmonary embolism (PE) detected post-mortem are neither suspected nor diagnosed before death [3]. The incidence of VTE rises during hospitalization as a result of increases in predisposing factors; reportedly, approximately 78% of hospitalized patients have more than one risk factor for VTE, and around 20% of patients have at least three risk factors [4]. The standard approach for the prevention of VTE is pharmacologic thromboprophylaxis with unfractionated heparin or low-molecular weight heparin [5].

National Institute for Health and Care Excellence guidelines state that all patients should receive verbal and written information on the risks and consequences of VTE and the potential adverse effects of thromboprophylaxis and risk-reduction strategies prior to starting thromboprophylactic treatment [6]. Increased patient awareness of VTE and thromboprophylaxis may promote patient safety by facilitating active participation in recommended activities such as early ambulation and calf-pumping exercises. Reportedly, patient refusal is the most common reason for missed doses of thromboprophylactic treatment [7]. Therefore, patient education prior to the start of treatment may improve adherence, especially when patients understand the purpose of their medication. In addition, it has been shown that education on the potential adverse effects of thromboprophylactic treatment is not necessarily associated with its refusal by patients [8]. Rather, it may increase patient recognition of serious adverse effects and promote fast reporting. Moreover, knowledge of the signs and symptoms of VTE helps patients to assess and report them during hospital admission and after discharge to obtain timely medical help, especially in those at high risk of VTE recurrence.

Although, a number of general reports have focused on the importance of preventing VTE [9–11], few studies have assessed patients’ awareness of VTE and their satisfaction with thromboprophylactic treatment, particularly in Arabic-speaking countries. Therefore, in this cross-sectional study, we evaluated patients’ awareness of VTE, perceptions of thromboprophylaxis, and satisfaction with the information provided on VTE and thromboprophylactic treatment.

## Methods

### Study design

In this cross-sectional study, we distributed a survey to adult patients hospitalized in medical wards at the King Abdulaziz Medical City (KAMC), Riyadh, Saudi Arabia, between December 2015 and March 2016. The KAMC is a 1200-bed tertiary care academic hospital accredited by the Joint Commission International. Patients were selected by convenience sampling, and were included in the study if they had received thromboprophylaxis (5000 units of heparin subcutaneously (SC) q8–12 h, 7500 units of heparin SC q12 h, or 30–40 mg of enoxaparin SC once daily). Patients were excluded if they were critically ill, admitted to the emergency department, receiving ambulatory care, or cognitively impaired. The average number of adult patients receiving thromboprophylaxis during the study period was 350. With a confidence level of 95%, margin of error of 5%, and response distribution of 50%, the minimum recommended sample size for this study was 184 according to the software Sample Size Calculator (Raosoft, Inc., Seattle, WA, USA). Patients were approached on the third day of admission to allow adequate exposure to any kind of education on VTE or thromboprophylaxis. Eligible patients were provided with a description of the study and its objectives, and were then asked to participate. Those who agreed to participate were interviewed by one of the researchers to ensure that all survey items were clear and comprehensive.

### Survey instrument

The survey was developed by combining two previously validated surveys [12, 13], and the questions were selected based on the study objectives. The survey was translated into Arabic and validated in a pilot study involving 43 Arabic-speaking participants. Analysis was also performed to assess the reliability of the combined survey.

The survey consisted of 18 closed-ended questions intended to discern:

1. demographic information including age, sex, level of education, and reason for admission;
2. personal or family history of VTE and thromboprophylaxis;
3. awareness of DVT and PE, including their underlying causes, risk factors, symptoms, and prevention;
4. perceptions of pharmacologic thromboprophylaxis and information received on VTE; and
5. satisfaction with thromboprophylactic treatment and related information received.

Responses to some of the survey items measuring awareness, perception, and satisfaction used a five-point Likert scale ranging from *strongly disagree* to *strongly agree*.

The content validity of the translated combined version of the survey was established by two expert individuals who examined the appropriateness of the content after making necessary modifications to items to ensure that they were sufficiently comprehensive and accurately assessed awareness of VTE and perceptions of thromboprophylaxis. In addition, the reliability of the survey was examined using Cronbach’s alpha (α), a measure of internal consistency that indicates how closely related the set of items are as a group.

The completed questionnaires were collected and safely stored in the principal investigator’s office. Data were saved into an appropriately designed Excel® spreadsheet (Microsoft Corp., Redmond, WA, USA).

Data were processed in accordance with the best practices for raw data management to identify any inaccuracies or omissions prior to statistical analysis. To accomplish this task, all interval variables were checked and summarized in terms of minimum and maximum values. These values were checked and compared against the possible minimum and maximum values of each variable, and variables with implausible values were flagged. A similar process was applied to categoric variables to identify any potential anomalies using a general frequency analysis.

### Statistical analysis

Descriptive statistics including the number and percentage of respondents by demographic characteristics and personal and family history of DVT and PE were calculated. Percentage awareness of correct and incorrect signs and symptoms of DVT and PE were computed. Percentage positive perception and satisfaction (indicated by a response of *agree/strongly agree*) were also calculated. Awareness of DVT and PE were compared by a number of characteristics including age, sex, level of education, and personal and family history of VTE using the chi-squared test. Statistical significance was set at *p* < 0.05. All statistical analyses were performed using SPSS (Release 21.0.0.0; IBM Corp., Armonk, NY, USA).

## Results

Out of 190 patients have been screened, 174 participants completed the questionnaire, constituting a response rate of 95%. Descriptive statistics for the respondents are displayed in Table 1. Fifty-six percent of respondents were aged over 50 years, and 52% were male. Most respondents had an educational level of high school or lower (77%). Sixty-three percent of respondents were admitted for medical treatment. Only 14 and 12% of respondents had personal or family histories of VTE, respectively. Most respondents (72%) were aware that they were receiving pharmacologic/non-pharmacologic thromboprophylaxis at the time of questionnaire completion, whereas only 31% reported a history of thromboprophylactic treatment.

**Table 1:** Profile of participants (*N* = 174)

Factor	Number	Percent
Age Category (years)		
18–30	32	18.4%
31–50	45	25.9%
51–70	66	37.9%
71+	31	17.8%
Gender		
Male	90	51.7%
Female	84	48.3%
Education Level		
Uneducated	65	37.4%
Less than high school	42	24.1%
High school	27	15.5%
University	32	18.4%
Higher education	8	4.6%
Reason for Admission		
Surgical	60	34.5%
Medical treatment	110	63.2%
Oncology (nonsurgical)	3	1.7%
Palliative care	1	0.6%
Personal History of VTE		
Yes	25	14.4%
No	143	82.2%
Unknown	6	3.4%
Family History of VTE		
Yes	21	12.1%
No	141	81.0%
Unknown	12	6.9%
Currently Receiving Pharmacological/non-Pharmacological Thromboprophylaxis		
Yes	126	72.4%
No	36	20.7%
Unknown	12	6.9%
History of Receiving Pharmacological/non-Pharmacological Thromboprophylaxis		
Yes	54	31.0%
No	111	63.8%
Unknown	9	5.2%

As shown in Table 2, only 32 and 15% of participants reported knowledge of DVT and PE, respectively. Of the respondents aware of DVT, 55% (30/55) identified swelling of the leg as a symptom. Other correct symptoms, including pain/tenderness in the leg, noticeable changes in the color of the leg, and noticeable changes in the temperature of the leg, were selected by 49% (27/55), 29% (16/55), and 18% (10/55), respectively. Incorrect signs and symptoms of DVT, including leg paralysis, itching of the leg, and others, were selected by 36, 20, and 15% of respondents, respectively. Relatively higher accuracy levels were observed in response to questions reflecting patient awareness of the signs and symptoms of PE, where correct answers including chest pain, shortness of breath, lightheadedness, and coughing up blood were selected by 69% (18/26), 69% (18/26), 23% (6/26), and 23% (6/26) of respondents, respectively.

**Table 2:** Awareness of VTE

Item	Options	No. of responses	Percent
Which of the following cause DVT?	Blood clot in vein^b^	82	47.1%
	Lack of oxygen in vein	5	2.9%
	A tumor in vein	7	4.0%
	None of the above	4	2.3%
	Not sure	76	43.7%
Know what a blood clot in your leg is or DVT?	Yes	55	31.6%
	No	119	68.4%
Which of following are signs/symptoms of DVT?^a, c^			
	Swelling of leg^b^	30	54.5%
	Itching of leg	11	20.0%
	Pain/tenderness in leg^b^	27	49.0%
	Noticeable changes in color of leg^b^	16	29.0%
	The leg feels warm^b^	10	18.1%
	Leg paralysis	20	36.3%
	Other	8	14.5%
	Not sure	2	3.6%
Know what a blood clot in your lung or PE?	Yes	26	14.9%
	No	148	85.1%
Which of following are signs/symptoms of PE?^a, d^			
	Shortness of breath^b^	18	69.2%
	Slow, shallow breathing	5	19.2%
	Chest pain (may be worse with deep breath)^b^	18	69.2%
	Rapid heart rate	7	26.9%
	Lightheadedness/passing out^b^	6	23.0%
	Pain radiating down arm	5	19.2%
	You cough up blood^b^	6	23.0%
	You have frequent headaches	4	15.4%
	Other	3	11.4%
	None of the above	1	3.8%
Which of the following might increase your risk of developing a blood clot?^a^			
	A hospital stay^b^	49	28.2%
	Surgery^b^	67	38.5%
	Cancer^b^	47	27.0%
	Not moving a long time^b^	119	68.4%
	Pregnancy/giving birth^b^	40	23.0%
	Using estrogen-based meds^b^	14	8.0%
	Family history of blood clots^b^	59	33.9%
	Older age (65+)^b^	72	41.4%
	Too much exercise	6	3.4%
	High blood cholesterol^b^	92	52.9%
	Donating blood	8	4.6%
	High blood pressure^b^	83	47.7%
	Other	24	14.1%
	None of the above	2	1.1%
	Not sure	41	23.6%
Which of following help prevent a blood clot?^a, e^	Walking/stretching legs^b^	76	92.7%
	Drinking plenty of fluids	37	45.1%
	Eating lots of fiber	41	50.0%
	Bed rest	11	13.4%
	Washing/bathing regularly	27	32.9%
	Don’t know	11	13.4%
	Other	9	5.2%

*N* = 174

^a^More than one response allowed

^b^Indicates correct response

^c^N = 52

^d^N = 26

^e^N = 82

Sixty-eight percent of respondents correctly identified “not moving for a long time” as a risk factor for developing a blood clot. However, other risk factors including hypocholesteremia and hypertension were correctly identified by about half of the respondents. Fewer respondents correctly selected other risk factors such as advanced age (41%), family history of blood clots (34%), and pregnancy/childbirth (23%). Most respondents were aware of the benefits of walking or stretching the legs for the prevention of blood clots (93%). However, a relatively high percentage of activities like drinking fluids (45%), eating lots of fiber (50%), and washing/bathing regularly (33%) were incorrectly identified as measures that may prevent blood clots.

**Fig. 1:**
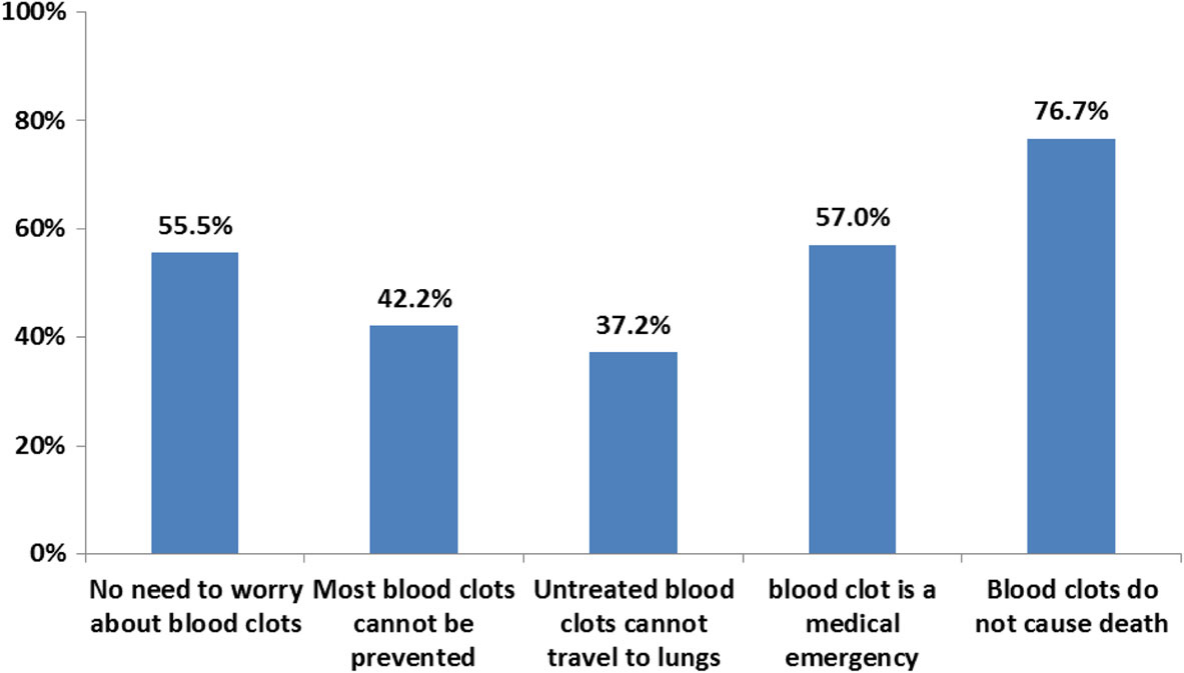
Awareness of VTE*. *Percent disagree/strongly disagree

The results for patient awareness as measured using a Likert scale (Fig. 1) showed that most respondents agreed that blood clots can cause death (77%), whereas just over half of them were aware that blood clots can develop at any age (56%), and are considered a medical emergency (57%). Only 42% of respondents thought that most blood clots can be prevented, and only 37% knew that they can travel to the lungs.

**Fig. 2:**
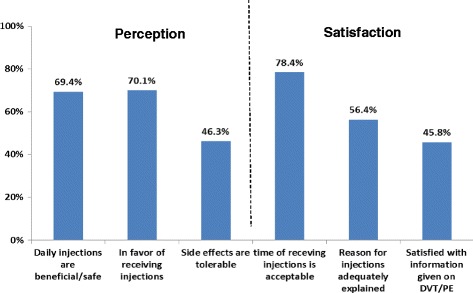
Perception and Satisfaction about Pharmacological Thromboprophylaxis and Information Received on VTE*. *Percent agree/strongly agree

Figure 2 shows that respondents generally had a positive perception of thromboprophylaxis: about 70% reported that they considered the treatment beneficial and safe and were in favor of receiving it. However, less than half reported that the adverse effects of the treatment were tolerable. Most respondents were satisfied with the time they received the treatment (78%), but only 56 and 46% were satisfied with the information they received about the treatment and about DVT/PE, respectively.

The results of analyses of respondents’ awareness of DVT or PE by age, sex, level of education, and personal and family history of VTE are shown in Table 3. The percentage of respondents reporting awareness of DVT or PE was significantly higher among those with a personal or family history of VTE: 68% versus 32%, *p* = 0.001, and 57% versus 35%, *p* = 0.046, respectively. Awareness of DVT was not associated with any of the other factors listed in the table.

**Table 3:** Number and percentage of respondents reporting awareness of DVT or PE by respondents’ characteristics

	N	Number aware	Percent	*p*-value^a^
All Respondents	174	65	37.4%	
Age Category (years)				0.18
18–50	77	33	42.9%	
51–70	97	32	33.0%	
Gender				0.61
Male	90	32	35.6%	
Female	84	33	39.3%	
Education Level				0.26
High school or below	134	47	35.1%	
University/Higher education	40	18	45.0%	
Personal History of VTE				0.001
Yes	25	17	68.0%	
No	149	48	32.2%	
Family History of VTE				0.046
Yes	21	12	57.1%	
No	153	53	34.6%	

^a^Based on the Chi-square test

## Discussion

The findings of our study indicate poor awareness of DVT and PE among hospitalized patients (32 and 15%, respectively). Correspondingly, they demonstrate the lack of awareness of the signs and symptoms of DVT and PE. This is consistent with the results of previous studies. A national survey conducted in the United States found that 74% of adults had a poor knowledge of DVT and its complications [14]. The lack of awareness of VTE is a common problem worldwide that is not limited to distinct patient nationalities or populations. Studies involving pregnant women, postnatal women, and cancer patients have reached similar conclusions [15–18].

Our study also shows that more than half of respondents were unaware of the causes of DVT and that 63% do not believe that blood clots can travel to the lungs. Such findings indicate that the consequences of blood clots and the link between DVT and PE are underestimated. The lower awareness of PE compared with that of DVT may explained by the pathophysiologic nature of PE; it is a life-threatening condition considered a complication of DVT, and it is often termed the “silent killer” because of the nonspecificity of its symptoms [19]. This has also been documented in other reports [12, 13].

Most participants who correctly identified risk factors for VTE recognized immobility as a key risk factor for DVT and PE development. This result may reflect the efforts of health-care providers to encourage hospitalized patients to ambulate. However, these efforts may result in insufficient patient education on the risks associated with other health conditions, as conveyed by reports on nurses’ experiences of the implementation of VTE prophylaxis [20]. A recent study on awareness of VTE during preoperative assessments reported that only 47% of patients had received verbal or written information on the condition, and that although many patients were aware of VTE, detailed information regarding its risk factors and prophylaxis was lacking [16]. This finding suggests the need to provide patients with more detailed information on VTE to ensure a better understanding of its risks and prevention.

Approximately half of patients incorrectly identified risk factors such emotional and psychologic trauma, exposure to cold air, or fever. Similarly, they were unaware of important risk factors such as cancer, which corroborates the findings of other studies in which awareness of cancer as a risk factor for VTE was poor among oncology patients [21, 22]. This information is crucial, because it helps patients to understand the rationale for thromboprophylaxis during cancer treatment; thus, cancer patients should receive more information. Our results also show that there is misperception of the signs and symptoms of DVT, as indicated by the confusion of DVT with other conditions such acute coronary syndrome (ACS). This was also noticed by interviewers when asking patients about VTE terms, and may be related to the similarity between the Arabic words for VTE and ACS. When comparing the participants in terms of the level of DVT or PE awareness, we found that demographic characteristics (age, sex, and educational level) had no impact. In contrast, awareness of DVT or PE was significantly higher among those with a personal or family history of VTE.

In this study, about 20% of respondents were unaware that they were receiving pharmacologic thromboprophylaxis, which may reflect the failure of health-care providers to provide patients with counseling in regard to their treatment during hospitalization. This may be related to respondents’ poor satisfaction regarding the information they received about pharmacologic thromboprophylaxis and its adverse effects. Most respondents, however, had a positive perception of pharmacologic thromboprophylaxis, and the majority agreed that the treatment was beneficial to their health and that they were in favor of receiving it.

Better education on VTE, DVT, and PE terms, risk factors, and preventive measures is needed to encourage active involvement by patients in treatment plans, ensure their adherence particularly after hospital discharge, and promote self-diagnosis and reporting of VTE symptoms. This effort should also be extended to the general public, because we observed that level of education was unrelated to DVT awareness. Educational campaigns can be beneficial and have proven effective in increasing public awareness of VTE [17].

Our study has a number of limitations. First, its small sample size limits our ability to generalize the study results beyond our institution. The total number of participants is smaller than the calculated sample size, as some patients were not available at time of patient screening. Nevertheless, our findings were consistent with those from a number of studies, including a study utilizing a global survey administered to the general public in nine countries: Argentina, Australia, Canada, Germany, Japan, Thailand, the Netherlands, the United Kingdom, and the United States [12–16]. Secondly, closed-ended survey questions may have helped respondents to guess rather than answer with regard to their knowledge, particularly in response to questions where more than one answer was allowed (the average number of answers per question ranged from four to eight). To minimize guessing, the interviewers attempted to ask the survey questions in an open-ended manner, to allow enough time for the participants to think before providing their answer.

## Conclusion

This study clearly demonstrates that awareness of VTE in general, and of DVT and PE terms—in particular, the risk factors, signs, and symptoms—among hospitalized patients is inadequate. More emphasis should be placed on the education of at-risk patients to promote adherence to treatment, self-diagnosis, recording of DVT and PE symptoms, and active involvement in safety. This study sets the stage for further research to determine the optimal approach to the education of hospitalized patients to promote safe and high-quality patient care. The findings of this study may also encourage health-care providers to deliver more education to patients and public health organizations about VTE, DVT, and PE, and their risk factors, signs and symptoms and preventive measures.

## Abbreviations

ACS: Acute coronary syndrome
DVT: Deep vein thrombosis
PE: Pulmonary embolism
SC: Subcutaneously
VTE: Venous thromboembolism
